# Possible Mechanisms of Hypnosis from an Interactional Perspective

**DOI:** 10.3390/brainsci11070903

**Published:** 2021-07-09

**Authors:** Katalin Varga

**Affiliations:** MTA-ELTE Lendület Adaptation Research Group, Institute of Psychology, ELTE Eötvös Loránd University, H-1064 Budapest, Hungary; varga.katalin@ppk.elte.hu

**Keywords:** interactional synchrony, corrective relational experiences, orbitofrontal cortex, oxytocin

## Abstract

In this paper, utilizing the interactional research paradigm developed by Éva Bányai, we discuss the hypnotic relationship from the viewpoint of interactional synchrony. Based on our three decades of empirical studies of an interactional paradigm, we propose the analogy between hypnosis and mother–child interaction. Hypnosis is considered as a potential corrective/reparative possibility when the real childhood experiences appear to be unfavourable. Possible neuroanatomical and neurochemical mechanisms are also suggested in the right hemispheric orbitofrontal cortex and central oxytocin system.

## 1. Introduction

Norcross [1] pointed out that at least a hundred studies have arrived at the uniform conclusion that clients in psychotherapy attribute the success of their treatment not to the technique or the method, but rather to their *relationship* with the therapist.

In our approach based on three decades of empirical studies in an interactional paradigm, we propose that current hypnotic interactions may be related to experiences connected with some previous—typically early childhood parent-child—relationship patterns. We further propose that interpersonal experiences under hypnosis may have a corrective effect especially if the person did not have the opportunity to experience profound attunement in his or her real relationships. In this paper a theoretical proposal is outlined: the parent–infant dyadic studies and models will be discussed along with the analysis of hypnosis interactions, with special emphasis on the potential neuroanatomical and neurochemical mechanisms. The reader is invited to study the details of the empirical foundation in the referenced papers.

The detailed observation of infants in interaction with objects and persons in their environment made it apparent that humans are interactional from the very beginning of life. Human infants, at only a few months of age are already attuned to the other human beings around them; not only are they able to communicate their own intentions and subjective states, but also demonstrate an ability to read the intentions of others and detect their subjective states (see, e.g., the concept of “intersubjectivity” of Trevarthen and Aitken [2]). These skills soon enable the infant to pay joint attention towards an external object coupled with another human being (cooperative or secondary intersubjectivity).

In the field of hypnosis, it is increasingly recognized in both the experimental and clinical areas, that there is a dynamic, two-way, unconscious communication between the hypnotists and their subjects [3]. However, there are only rudimentary ideas about its mechanism as of now.

Norcross lists several names for the degree of fitness between the two partners in psychotherapeutic situations: “responsiveness, customizing, attunement, tailoring, matchmaking, aptitude by treatment interaction” [1] (p. 126). The quantity of these concepts reflects that many authors observed—and named in various forms— this interactional phenomenon. It is recently most commonly called “interactional synchrony”. Waugh [4] argued that the concept of interactional synchrony is more dimensional than dichotomous: It can appear in various degrees. It is advantageous if both the presence or the absence of harmony (or as Waugh said: interactional harmony and discord) can be handled within the same theoretical model and empirical approach. In this dimensional framework, it is also easier to study what kind of synchrony or harmony is optimal in each situation. Most authors agree that a constantly high level of interactional synchrony is undesirable. The goodness of fit model [5] says that optimality is determined by how well the demands of the partners fit their possibilities.

The optimal level of coordination with the interactional partner is important, because this is what promotes the experience of finding identity with and distinction from the others in the best way, as Brewer’s [6] model of optimal distinctiveness states.

Most of the studies that of the interaction between small children and their parents are based on the observation of behavioural data. The researchers determine the degree or pattern of synchrony based on these recordings. The methods applied in our studies however—enabled us to ask directly how the interactants rated the interaction itself, and to infer the above-mentioned fitness.

## 2. Method and Main Results

In our studies of hypnotic dyads, the signs of fitness and tension clearly appeared in indices of experience measured by the Phenomenology of Consciousness Inventory (PCI) ([7,8] and some subscales of the Dyadic Interactional Harmony (DIH) [9]. The PCI taps the personal subjective feelings of the participants, while DIH measures the way they evaluate their interaction itself. The joint analysis of the two members of the dyad made it possible to infer possible mismatch reflected by the sharp contrast between the judgments of the two persons (see Table 1 for a summary of our main findings). In this way the degree and nature of interactional synchrony seems to be measured easily in the case of dyadic hypnotic interaction.

## 3. Role of Mother–Baby Interaction in Early Development

In his analysis, Schore [22] localized the internalized working models that fix the schemata of emotional regulation, that also have a cognitive-affective charge, to the fronto-limbic orbital cortex of the right cerebral hemisphere.

This is the region that takes part in the perception and interpretation of species-specific facial expression, prosody (affective tone and emotionally expressive voice), and gestures, in the regulation of emotional behaviour, analysis of the emotional charge of environmental stimuli, and the mental generation of internal images and facial expressions. In the end, this region of the cortex plays a mediating role between the internal world and the environment in the affective-motivational sphere.

In the region of the fronto-limbic orbital cortex of the right cerebral hemisphere, we have a chance of inducing changes through the corrective relational experiences (as we will explain in greater detail later):

“…The relationship is key. It is through the experience of seeing and hearing into the patient—of creating an experience of resonance inside oneself, of listening from within where the patient is—that one can begin to realize attunement, to the nuances, rhythms, language and emotional movements of the person who sits in the room with us. And it is through this attunement that we can help create the new neurological pathways, between frontal lobes and limbic system, for emotional regulation” [23] (p. 675).

According to Shore [22] the analysis of the stimulus input—in infancy, typically the face and the prosodic features of the voice of the mother—is organized into a uniform whole. It is interpreted primarily from the aspect of emotional tone and then that of matching motivational tendency in the multimodal association areas of the temporal and frontal cortices. In the course of evaluation, the individual matches the characteristics of the incoming (current) stimulus to the templates of the emotional-expressive patterns stored in the right hemisphere. If the stimulus gets a positive evaluation (the face and voice of the mother), the original stimulus is amplified by the activation of the subcortical units in the infant, eliciting a general “pleasant emotional state”, activating the tegmental reinforcement system and increasing arousal. From here, through the hypothalamus, the motivational and action (behavioural) tendencies appropriate for the stimulus (and the arising emotional state) are also activated. The maturation of this system—and especially that of the orbitofrontal cortex in it—is greatly facilitated by the mutual gaze and prolonged eye contact with the mother in the second half of the first year of life.

Schore [22] argues that if the mother does not fulfil this role of arousal-regulation, either because she is absent, or because she is present, but does not provide sufficiently meaningful stimuli (face, voice) for the developing nervous system of the infant, or causes dysregulation in the infant by overstimulation, the infant will often react with uncontrollable burst of anger towards the situation incomprehensible of them. In this case, the left hemisphere will be functionally separated from the unconscious self- and object-schemata in the right hemisphere, because they contain threatening and unmanageable content. As a result, among other things, the experienced emotional states will be unavailable for the analytical, verbal labelling and meaning attributing functions of the left hemisphere.

Based on all this, we can see that Holmes’s statement is not an exaggeration: “an individual’s unique bibliography becomes embedded in the structural biology of the brain” [24] (p. 170, emphasis added).

According to Shore, when the infant is about two years old, the previously mostly positive maternal emotions are supplemented by situations accompanied by the disapproving facial expressions of the mother. The mother expresses that she disapproves of something that the infant enjoys doing. First, the infant is surprised by this misattunement, for he enjoyed what he originally did. The disapproving feedback from the mother quickly reduces the arousal of the child, and through this mechanism, differential learning begins, in the course of which the child learns, by way of feedback from the social partners, what is socially expected and recommended, and what is not. This change corresponds to the state of “shame”, maternal feedback produces a state of inhibition and withdrawal, and the former sympathetic-ergo tropic activity suddenly changes into parasympathetic-trophotropic arousal [25].

## 4. Hypnotic Interaction and Early Mother–Infant Relationship

It may bring us closer to understanding hypnotic interaction if we consider the mutual attunement observed in early mother–infant relationships. Gruzelier [26] highlighted the role of the right hemispheric orbitofrontal cortex in this respect, an area that is known as the centre for social adaptation and emotional-motivational attunement. This area, that develops faster and is anatomically larger in the right hemisphere, plays a key role in the attachment process and in the organization of biological synchrony between mother and infant in the early years of life (see also [27,28,29,30]).

Adult attunement—in the course of therapy, for example—is not inherently cognitive, but emotionally charged; it is usually nonverbal, and—as Gruzelier puts it—it takes place “spontaneously”. Again, the mediating role of the right hemisphere—the limbic system in particular—seems to be decisive in this.

In Schore’s [25] analysis it was the front limbic orbital cortex of the right hemisphere that is responsible for the cognitive-affective working models and for the schemata of emotional regulation. Several phenomena of hypnosis can also be linked to the dominance of the limbic system in the right hemisphere [26]. This could be the neuroanatomical link of hypnosis providing corrective experiences.

### Attunement in Hypnotic Interaction

What could be the mechanism of the mutual attunement of hypnotist and subject? What modalities and channels connect the partners in hypnosis interaction?

Narrowing the attention of the subject during hypnotic induction gradually leads the subject to partially relinquish control to the hypnotist; from this point on, the hypnotist consciously begins to form the subjective reality of the subject. On one hand, the hypnotist guides the world of experiences of the subject directly, but on the other hand, the hypnotist also follows what takes place in the subject with intensive attention, and from the position of profound attunement s/he facilitates the development of a common background of interpretation and meaning. An outstanding example of this process is Visual Imaginative Synchrony (VIS), where the match appears between the visual experiences of the two persons [31].

During hypnosis, the verbal suggestions work on the—mostly—visual experiences of the subject, and they can be in parallel with real experiences, “The acceptance or translation of verbal content (hypnotic command) into experienced reality, an impressive dominance of concept over perception” [32] (p. 172).

If the patient—either in hypnosis or afterwards—describes his experiences in words, too, he must transfer them into a new modality, to the verbal world.

“In psychotherapy the patient uses words to describe an essentially visual record of an earlier experience” [33] (p. 111, emphasis added). During this, the patient must follow the rules required by verbality, by grammar. The expression itself has many advantages:

“One learns and becomes more fully, who one is within conversation with another. In the process of speaking—or signing, or picturing, whatever form of the process of symbolic communication—one hears aloud one’s thoughts outside oneself, and thus one can hear in a different way, walk around the thoughts, view them from different angles, and absorb, comprehend and amend them. When speaking occurs, with/to another, who is truly listening and absorbing and reflecting what she is hearing, then what is inside us achieves even fuller dimensionality through crystallizing, in concrete, visible form, the actuality of thought itself. What was once unheard, unfelt thought now reaches visible, sensorially experienced impact and response from the other” [23] (pp. 663–664).

The relationship between verbal and visual modality also appears in a reverse direction. In the case of hypnosis, we can influence the generally visual experiences (images) of the subjects/patients by the (generally) verbal methods of suggestions. It is key in bringing these modalities—i.e., visual or verbal—together that both are organized along a determined system of rules.

“There is an inherent connection between language, imagery, and movement in that they all require grammar and structure. These are all aspects of communication, which may be the essence of the hypnotic session” [32] (p. 172).

The key question is how these inner processes of imagination find their way to reality. This will be discussed in the next section.

## 5. Mental Imagery and Subjective Reality

Some of the cortical representations have a dual function: They serve both perception and imagery. Mental imagery is represented in the visual system. Furthermore, various characteristics, such as colour, shape, spatial arrangement are represented distinctly in both cases [34]. Consequently, the imaginative attunement caught in the cases of visual imaginative synchrony [31] may give an experience identical to real perceptual experience.

Putting it more broadly: imagery drawn by hypnotic suggestions (including all its modalities) may provide an experience of subjective reality that is such as objective reality. Therefore, it is important that the subject’s eyes are closed during (relaxational) hypnosis: This way, the perceptual traces of the external, objective reality do not conflict with the internal, subjective processes.

The possibilities offered by imagery are almost limitless. In order to correct a disturbed pattern of attachment, the patient can be led through the whole process of development in the imagination, from the experience of the infant snuggling in its mother’s arms, through watching its hands wandering in its visual field, and through the experiences of the distinction between me-and-the other to exploring the external world, and so on [35]. In this working mode reality can be “bypassed”—of which several examples are given in [18]—either by building in corrective experiences that do not match historical reality, or by preserving objective reality, but the outcome depends on the events experienced in the imagination.

## 6. Vegetative and Neurohormonal Background of Cognitive and Affective Regulation on Social Context

Porges [36,37,38] developed a theoretical model that connects autonomic (vegetative) regulation and social behaviour. According to his theory, the appearance of the behaviour and mental states related to affiliation, parenting, and intimacy are related to the emergence of the polyvagal system in evolution. In the course of evolution, the system that enables fast changes in accordance with the momentary situation—in addition to the simpatico-adrenergic control of heart-rate regulation—first appeared in mammals. This made complex forms of behaviour possible—such as attention, orientation, relaxed state, learning, and social engagement—that are indispensable for the development of social behaviour and attachment. Consequently, sufficiently fast, and fine-scale adaptation to the subtle changes of social contact—emotional facial expression, direction of gaze, subtle gestures, etc.—could develop. The model of Porges has been criticized as lacking appropriate empirical basis, nevertheless it is one of the most well-known models regarding the role of vegetative system in the regulation of social behavior in mammals.

Feldman [39] and her group recorded the heart rates of 3- and 6-month infants and those of their mothers simultaneously; they found that these rates attuned to each other gradually. The precondition for this was enough gaze synchrony. If mutual gaze was missing—typically in depressed mothers—the harmony between the heart rates could not be realized. According to Feldman’s [40] studies, the careful adaptation of the mother to the social stimuli of the infant in mother-and-infant interactional synchrony situations regulates the heart rate of the child. It is hypothesized that this is experienced and internalized by the infant as the emotional experience of security, which remains with the person for the rest of his/her life.

Oxytocin (OT), as a neurotransmitter—apart from its peripheral role during delivery and breast feeding—is a key component of psycho-affective regulation throughout our whole life, as the central oxytocin effect [41,42,43].

It has been demonstrated that early neglect or abuse is accompanied by lower levels of OT [44], and in cases of early neglect, decreased OT responses are present to social support, compared to non-neglect cases [45]. There are many examples of the decisive role of the quality of maternal care: overprotective, emotionless, controlling maternal styles are risk factors in depression, antisocial personality structure, anxiety disorder, drug-abuse, and compulsive and attentional disorders of the offspring [46]. Persons receiving neglectful maternal care are also more neglectful with their own offspring, and if they have been victims of abuse, they are also more likely to expose their children to abuse. Ambivalent attachment style leads to unfavourable consequences in children [47,48].

The support of the OT system through corrective experiences is significant, because with respect to the OT effects on memory, it has been proven that OT facilitates forgetting difficult/traumatic social experiences while activating the unconscious network of social memories [45]. The ultimate aim is the balance between the ancient defence system activated as a result of threat and uncertainty and the more recent attachment system built on the experiences of proximity, trust, and care in a social context.

## 7. Hypnosis Interaction and the Oxytocin System

Our results are concordant with the above-mentioned findings. We could see in our oxytocin data that the changes in the oxytocin level of the hypnotists were related to the emotions of the subject received from their parents [20]. In the psychogenetic study, we found that the relationship patterns of the scores on the Archaic Involvement Measure (AIM) and the Dyadic Interactional Harmony (DIH) differed as a function of genetic background. While the AIM was associated with genotypes connected to the dopamine system (the additive effect of the G allele of the catechol-O-methyltransferase (COMT) gene led to higher dependency need on AIM), DIH—evaluating the current hypnotic interaction—was associated with the serotonin system (carriers of short 5-HTTLPR allele scoring significantly higher on the Dyadic Interactional Harmony (DIH) intimacy scale compared to the long carriers) (for details see [18]. In connection with this, it is worthwhile to emphasize the relationship between hypnosis style [16] and interactional experiences (DIH): In the cases of maternal style hypnosis (when there is an emotionally comforting, positive atmosphere, as opposed to paternal style, that is based on respect and authority)—there was no correlation between DIH scores and hypnosis style scores in the subjects, but the same correlations were high in the hypnotists. The more maternal the style of hypnosis, the higher the level of intimacy. Apparently, the hypnotist provides the possibility for the subject to have a relationship experience by or along his or her own interactional experience-pattern.

### Hypnotizability: What Are the Lows High in?

Studies employing traditional hypnosis found no correlation between changes in oxytocin (OT) and hypnotizability [20] while during active-alert hypnosis (AAH) we were able to isolate three groups. The OT level of high hypnotizable subjects decreased during AAH, while increased in low hypnotizables and did not change in the medium hypnotizable group. These results seem to offer an explanation as to why subjects who do not show a lot of overt behavioural response to hypnosis (that is why they are called “lows”) can still benefit from it and why many clinicians do not put much weight on the hypnotizability of their clients [21].

The phenomenological data (PCI) of AAH showed that increased OT related to lower altered state of awareness, concentration and vividness of imagery, higher self-awareness, less distorted time sense, and perception. These results also showed that lower behavioural phenomenological involvement does not necessarily mean that these subjects are not able to mobilize their OT system and profit from this special social setting [21].

## 8. Hypnosis: Modelling of Relational Patterns

In the twin study [13] (see also Table 1), we found that the experiences rating the hypnotic interaction (DIH) of monozygotic twins were correlated more with each other than with their actual interactional partners (the hypnotists). This harmony was not present in their behavioural (hypnotizability scores) or phenomenological (PCI) response-patterns.

These results can well be interpreted as related to the early interactional patterns, while the hypnosis situation can be seen as providing the opportunity of activating (bringing to the surface) early relationship patterns—in accordance with the predictions of the social psychobiological model of hypnosis [15,16,49].

Thus, there are many signs permitting the proposal that today’s hypnosis can reach back to the relational patterns of decades ago. However, there is a possibility to analyse this process in a broader perspective, too. It can have a transgenerational effect on how an individual experiences a relationship. The way a parent reacts to a child may depend on how the parent was reacted to as a child. Growing up with a responsive, accepting parent increases the likelihood that we will also react sensitively and empathically to our own children [50,51]. In less favourable cases—when the parental pattern was neglecting and not sensitive enough—the provision of corrective experiences may set the affiliative processes on a better path not only for the given person, but for his/her offspring, too.

The therapeutic procedures affecting the oxytocin system can offer a possibility to restore the resistance to stress and the favourable operation of social relationship systems by compensating for the consequences of early losses [52,53]. From a theoretical point of view, hypnosis, representing both verbality and imaginative working mode, may be a special tool in the hands of the clinician in approaching and/or working through transgenerational memories or even fantasies. The relational experiences within psychotherapy of appropriate atmospheres give a chance for the modification of the pattern of representation of important relationships [54]. The patient may get a corrective experience in hypnosis, in this extremely enhanced emotional mutual attunement, when the hypnotist perceives, understands, and appropriately responds even to his/her unexpressed feelings [16,17].

Systems involved in socioneurobiology of affiliation are flexible and able to assimilate novel information [55,56]. According to our results, changes in oxytocin-level—which is related to the dopaminergic system—appeared in the hypnotist, who was in the “caretaker” position, as a function of how the subject perceived his/her parents’ emotional warmth.

As it is well known, working models develop on the foundations of early relational patterns [57] affecting many areas in adulthood: how valuable the person feels himself to be, what kind of behavior he can expect from others and shows if one is or is not worthy of love. Working models may only be modified upon appropriate social effects.

In our study independent judges could reliably identify maternal and paternal hypnosis styles based on the video recordings of the sessions [19]. In hypnosis experiments, subjects can also rely on most of these open behavioural characteristics—e.g., smiling, touch, eye contact, words used, calling the subjects by their first name (for more details see, e.g., [49]). These may signal the subject what kind of relational pattern can be expected in the given hypnosis, which patterns would possibly be mobilized (or consciously recalled or visualized by way of fantasy) along which the subject can organize his/her interactional expectations. We hypothesize that the subject also communicates this to the hypnotist; therefore, a kind of “interactional bargaining” may develop. If the “supply and demand” sides get closer and closer to each other, we will probably find synchronous phenomena in the experiences of the participants, and a nice pattern of mutual attunement will develop. If bargaining remains one-sided and unsuccessful, the experiences of the interactants will probably go side by side, and we will see cases of disharmony or independence.

This exactly matches the dynamic, mutually embedded, circular, and mutually active process that is described by the concept of enactive intersubjectivity [58,59]. This conceptualization looks at social cognition as a mutual, active, dynamic process between the interacting partners (as opposed to the representational conceptualizations which describe an individual who observes the other from the position of a “third person”, and who tries to understand, map, and represent the mental state of the other).

According to the model of enactive intersubjectivity, it is the very interaction that gives meaning to the situation that is mutually formed by both parties, in which both participate with their whole identities, and in which they have a continuous effect on each other. The rare moments when the developing meanings fit each other perfectly are the very moments of “subjective” interactional synchrony. These statements apply to the hypnotic interactions, too.

## 9. Discussion: Hypnosis as a Corrective Relational Experience

Essentially, psychopathology can be considered as a limited capacity for stress-regulation; in these cases, the internal reparatory processes of interpersonal situations are deficient. These functions can be localized to the fronto-limbic area of the right hemisphere [25].

In the course of psychotherapy, the natural course of development is followed, but with the provision of a corrective example, building adaptive and productive patterns of solutions. The relationship with the therapist provides an opportunity to identify and evaluate the working model developed through the attachment to the early primary caretaker, and then to transform it in a corrective way. This is secured by the relationship with the therapist, which is the basis of correction by providing the pattern of secure attachment.

In order to achieve this, therapist and patient must deeply attune to each other emotionally, when the therapist resonates empathically to the emotions of the patient [60]. This deep relationship may evoke archaic relationship patterns in the patient—just as it was measured in our studies by the Archaic Involvement Measure with healthy subjects—and the correction of the developmental line stalled in real early development gets a chance.

In a similar vein, Peebles [23] describes the process as the therapist lets the signals from the patient “go through” him, enabling him to give meaning to them to reflect them back:

“It is helpful to listen carefully as the patient speaks—to open oneself to the experience of the patient’s speaking, momentarily toning down the precise tracking of each word. When rapid (even lively seeming) verbal agility does not stir engagement in the therapist, but instead incurs a sort of numbing and dissociation, or an experience of feeling overwhelmed, then the therapist may be experiencing a sophisticated manifestation of the patients’ dissociation” [23] (p. 654).

All this is topped by the therapist attaching words to the feelings, making it possible to put things the patient has never received emotional resonance to, not to talk about verbal labels, into words.

This is the experience the patient did not have, or only ambiguously, in the period of early attachment. His basic experience is that he can count on nobody in a stressful situation, the caregiver is not present, or if she is, she is not sensitive and responsive. Thus, the child learns that in tense situations he is left alone; many authors link this to the feeling of shame (see [25,61,62] on this topic). This is the pattern the person brings, this what he “uses” even decades later: He is not looking for a partner or relationship to solve or treat similar situations. The experience of shame calls for the need for hiding, and is accompanied by the inhibition of emotional expression.

The provision of corrective experience is sometimes rather concrete in the hypnotherapeutic practice with psychotics of Murray-Jobsis [35]. In the so-called nurturing technique, the patient in hypnosis may re-experience as his mother rocks him, looks at him, smiles at him, and holds him in her arms safely. In the method of creative self-mothering, positive parental care is provided to the child-self of the patient through the visualization of being simultaneously both a child and an adult, the therapist often representing the other parent. It is possible that in less specific situations, only the pattern of experiences brought about simply by the hypnotic situation may have the same effect (and perhaps may have a healing effect in less severe disorders).

Baker and Nash [63] argue that “the flesh and bone of psychoanalytic therapy is the therapeutic relationship itself” (p. 447). The hypnotic relationship has different roles as a function of the problem and treatment-type. In an insight-oriented situation, the appropriate “containment” helps the patient—through the positive aspects of working relationship and transference—to relax the defence mechanisms and to express and work through the conflictual material. In intensive, long-term therapies, the aim is to experience and tolerate the therapeutic relationship itself, and to identify the emotions arising in the relational experience. In some cases, the patient having elementary sensations or feelings may have the experience that the expression and sharing of these feelings can be tolerated, and can even be comforting in an appropriate interpersonal atmosphere [63]. This may sometimes mean a re-construction from the foundations, where the patient experiences his identity as an integrated whole from the elements of bodily experiences “within the holding context of therapeutic alliance and the continuity of the therapist’s emphatic and observational presence” [63] (p. 455).

The interactive nature of hypnosis is clearly formulated by some authors, e.g., by Baker [64]:

“These curative aspects of hypnosis work occur between the patient and therapist, not inside of the patient alone. These mutative experiences are the result of an interactive process that is formed by both patient and therapist as they influence one another reciprocally and jointly contribute to the evolving experiences of how they engage, relate, and construe the meaning of their relatedness. This interaction effect may well be the central mechanism for the therapeutic action of hypnosis. It is not what the hypnotist does which is critical. It is how the dyad construes and experiences how they are together that seems most evocative and evolutionary” (p. 65, emphasis added).

As a conclusion we can state that—although we are far from fully understanding the hypnotic interaction—the interactional approach to hypnosis and the detailed analysis of phenomenological data of both participants along with the neuroanatomical and neurohormonal changes seem to be a promising way to discover the real essence of hypnosis. From a general point of view: hypnosis, as a social interaction that can be standardized or freely varied, can be an ideal situation to study the neurophysiological processes of bidirectional influence of human dyads [65]. The above outlined “Model of Corrective and Interactive Hypnosis” is proposed to understand the dynamic interaction between the participants of hypnosis interaction. A deeper understanding of the corrective potential of hypnosis could lead to the increase of its therapeutic power.

## Figures and Tables

**Table 1:** Our main studies in phenomenology of hypnosis interaction.

The Focus of the Study	Main Finding	Reference
The relationship between the behavioural and the Subjective indices of hypnosis	The behavioural side of hypnosis and the experiences regarding the interaction itself showed no consistent concordance, especially if the interaction itself was evaluated	[10]
The relationship between the degree of phenomenological synchrony and hypnotic susceptibility	No consistent (linear) correlation was found between hypnotic susceptibility as measured by behavioural indices and the indices of interactional synchrony	[11,12]
The relationship between phenomenological patterns and the level of kinship	As opposed to behavioural scores—phenomenological indices are related to the level of kinship. The correlation between the evaluations given by monozygotic twins about the hypnotic interaction was very high, although they participated in two different hypnosis sessions with two different hypnotists at the same time	[13]
The relationship between phenomenological patterns and hypnosis styles	Characteristic relationships between phenomenological data and the construct of “hypnosis styles” (maternal and paternal hypnosis styles [14,15,16],). Both maternal and paternal styles enable the subjects to experience an altered state of consciousness; however, the more maternal the hypnosis, the more it is characterized by an experience of harmony and intimacy and by (both positive and negative) the emotional charge of the participants. In paternal style, the opposite was seen.	[17,18,19]
The level of oxytocin (OT) and cortisol in the hypnotic interaction: traditional relaxational hypnosis	The changes in OT is not related to the hypnotic susceptibility of the subject, rather, it shows a relationship with the relational experiences. After the hypnotic interaction, the level of oxytocin increases in the subject if the perceived harmony with the hypnotist is high, while it increases in the hypnotist if the subject has memories of less warm emotional relationships with his/her parents.	[20]
The level of oxytocin and cortisol in the hypnotic interaction: active alert hypnosis	The OT level increases compared to the baseline (before hypnosis) following the session. Typically, low hypnotizable show this pattern.	[21]

## References

[1] Norcross J.C., Duncan B.L., Miller S.D., Wampold B.E., Hubble M.A. (2010). The therapeutic relationship. The Heart and Soul of Change: Delivering What Works in Therapy.

[2] Trevarthen C., Aitken K.J. (2001). Infant Intersubjectivity: Research, Theory, and Clinical Applications. J. Child. Psychol. Psychiatry.

[3] Peebles-Kleiger M.J. (2001). Contemporary psychoanalysis and hypnosis. Int. J. Clin. Exp. Hypn..

[4] Waugh R.M. (2002). A Grounded Theory Investigation of Dyadic Interactional Harmony and Discord: Development of a Nonlinear Dynamical Systems Theory and Process–Model. Ph.D. Thesis.

[5] Hane A.A., Feldstein S., Dernetz V.H. (2003). The Relation Between Coordinated Interpersonal Timing and Maternal Sensitivity in Four-Month-Old Infants. J. Psycholinguist. Res..

[6] Brewer M.B. (1991). The social self: On being the same and different at the same time. Personal. Soc. Psychol. Bull..

[7] Pekala R.J. (1991). The Phenomenology of Consciousness Inventory.

[8] Pekala R.J., Steinberg J., Kumar V.K. (1986). Measurement of phenomenological experience: Phenomenology of Consciousness Inventory. Percept. Mot. Ski..

[9] Varga K., Józsa E., Bányai É.I., Gősi-Greguss A.C. (2006). A New Way of Characterizing Hypnotic Interactions: Dyadic Interactional Harmony (DIH) Questionnaire. Contemp. Hypn..

[10] Varga K. (2013). A Hipnózis Viselkedéses és Szubjektív Mutatói [The Relationship between the Behavioral and Subjective Indices of Hypnosis]. Ph.D. Thesis.

[11] Varga K., Józsa E., Bányai É., Gősi-Greguss A.C., Koester G.D., Delisle P.R. (2009). Patterns of interactional harmony: The phenomenology of hypnosis interaction. Hypnosis Theories, Research and Applications.

[12] Varga K., Józsa E., Bányai É.I., Gősi-Greguss A.C. (2012). Phenomenological synchrony and hypnotic susceptibility. Contemp. Hypn. Integr. Ther..

[13] Varga K., Bányai É.I., Gősi-Greguss A.C., Tauszik K. (2013). Phenomenological aspects of hypnotic interactions: The effect of kinship. Int. J. Clin. Exp. Hypn..

[14] Bányai É.I., Gősi-Greguss A.C., Vágó P., Varga K., Horváth R., Van Dyck R., Spinhoven P., Van der Does A.J.W., Van Rood Y.R., De Moor W. (1990). Interactional Approach to the Understanding of Hypnosis: Theoretical Background and Main Findings. Hypnosis: Current theory, Research and Practice.

[15] Bányai É.I. (1998). The interactive nature of hypnosis: Research evidence for a social-psychobiological model. Contemp. Hypn..

[16] Bányai É.I., Hoogduin C.A.L., Schaap C.P.D.R., de Berk H.A.A. (2002). Communication in different styles of hypnosis. Issues on Hypnosis.

[17] Varga K., Bányai É.I., Józsa E., Gősi-Greguss A.C. (2008). Interactional Phenomenology of Maternal and Paternal Hypnosis Styles. Contemp. Hypn..

[18] Varga K. (2013). The Phenomenology of Hypnotic Interactions.

[19] Varga K., Kekecs Z. (2015). Feature-based coding system: A new way of characterizing hypnosis styles. Int. J. Clin. Exp. Hypn..

[20] Varga K., Kekecs Z. (2014). Oxytocin and cortisol in the hypnotic interaction. Int. J. Clin. Exp. Hypn..

[21] Kasos E., Kasos K., Pusztai F., Polyák Á., Kovács K.J., Varga K. (2018). Changes in Oxytocin and Cortisol in Active-Alert Hypnosis: Hormonal Changes Benefiting Low Hypnotizable Participants. Int. J. Clin. Exp. Hypn..

[22] Schore A.N. (2003). Affect Disregulation and Disorders of The Self.

[23] Peebles M.J., Nash M.R., Barnier A.J. (2008). Trauma-related disorders and dissociation. The Oxford Handbook of Hypnosis.

[24] Holmes J. (1996). Attachment, Intimacy, Autonomy. Using Attachment Theory in Adult Psychotherapy.

[25] Schore A.N. (1994). Affect Regulation and the Origin of the Self: The Neurobiology of Emotional Development.

[26] Gruzelier J.H. (2006). Frontal functions, connectivity and neural efficiency underpinning hypnosis and hypnotic susceptibility. Contemp. Hypn..

[27] Feldman R. (2015). Sensitive periods in human social development: New insight from research on oxytocin, synchrony and high-risk parenting. Dev. Psychopathol..

[28] Gordon I., Zagoory-Sharon O., Leckman J.F., Feldman R. (2010). Oxytocin and the Development of Parenting in Humans. Biol. Psychiatry.

[29] Swain J.E., Lorberbaum J.P., Kose S., Strathearn L. (2007). Brain basis of early parent-infant interactions: Psychology, physiology, and in vivo functional neuroimaging studies. J. Child. Psychol. Psychiatry.

[30] Swain J.E., Kim P., Spicer J., Ho S.S., Dayton C.J., A Elmadih A., Abel K.M. (2014). Approaching the biology of human parental attachment: Brain imaging, oxytocin and coordinated assessments of mothers and fathers. Brain Res..

[31] Varga S.K., Varga K. (2009). Visual Imaginative Synchrony. Contemp. Hypn..

[32] Jasiukaitis P., Nouriani B., Hugdahl K., Spiegel D. (1997). Relateralizing Hypnosis: Or, have we been Barking Up the Wrong Hemisphere?. Int. J. Clin. Exp. Hypn..

[33] Covino N.A. (1997). The integration of clinical and experimental work. Int. J. Clin. Exp. Hypn..

[34] Farah M.J., Gazzaniga M.S. (2000). The neural bases of mental imagery. The New Cognitive Neurosciences.

[35] Murray-Jobsis J., Rhue J.W., Lynn S.J., Kirsch I. (1993). The borderline patient and the psychotic patient. Handbook of Clinical Hypnosis.

[36] Porges S.W. (2001). The polyvagal theory: Phylogenetic substrates of a social nervous system. Int. J. Psychophysiol..

[37] Porges S.W. (2003). Social Engagement and Attachment: A Phylogenetic Perspective. Ann. N. Y. Acad. Sci..

[38] Porges S.W. (2007). The polyvagal perspective. Biol. Psychol..

[39] Feldman R. (2007). Parent-infant synchrony and the construction of shared timing; physiological precursors, developmental outcomes, and risk conditions. J. Child. Psychol. Psychiatry.

[40] Feldman R. (2007). Parent-infant synchrony biological foundations and developmental outcomes. Curr. Dir. Psychol. Sci..

[41] Lee H.J., Macbeth A.H., Pagani J.H., Young W.S. (2009). 3rd. Oxytocin: The great facilitator of life. Prog. Neurobiol..

[42] Uvnäs-Moberg K. (1998). Oxytocin may mediate the benefits of positive social interaction and emotions. Psychoneuroendocrinology.

[43] Uvnäs-Moberg K., Arn I., Magnusson D. (2005). The Psychobiology of emotion: The role of the oxytocinergic system. Int. J. Behav. Med..

[44] Heim C., Young L.J., Newport D.J., Mletzko T., Miller A.H., Nemeroff C.B. (2008). Lower CSF oxytocin concentrations in women with a history of childhood abuse. Mol. Psychiatry.

[45] Macdonald K., Macdonald T.M. (2010). The peptide that binds: A systematic review of oxytocin and its prosocial effects in humans. Harv. Rev. Psychiatry.

[46] Champagne F.A. (2008). Epigenetic Mechanisms and the Transgenerational Effects of Maternal Care. Front. Neuroendocrinol..

[47] Barnett S., Buckroyd J., Windle K. (2005). Eating disorders from parent to child: Mothers’ perceptions of transgenerational effect. Couns. Psychother. Res..

[48] Ward A., Ramsay R., Turnbull S., Steele M., Steele H., Treasure J. (2001). Attachment in anorexia nervosa: A transgenerational perspective. Br. J. Med. Psychol..

[49] Bányai É.I., Peter B., Bongartz W., Revenstorf D., Butollo W. (2002). Hypnosis and mainstream psychology. Munich the 15th International Congress of Hypnosis. Hypnosis International Monographs.

[50] Brethelton I., Munholland K.A., Cassidy J., Shaver P.R. (1999). Internal working models in attachment relationships. A construct revisited. Handbook of Attachment. Theory, Research and Clinical Applications.

[51] Taylor G.J., Bagby R.M., Parker J.D.A. (1997). Disorders of Affect Regulation.

[52] Nagasawa M., Kikusui T., Onaka T., Ohta M. (2009). Dog’s gaze at its owner increases the owner’s urinary oxytocin during social interaction. Horm. Behav..

[53] Meinlschmidt G., Heim C. (2007). Sensitivity to intranasal oxytocin in adult men with early parental separation. Biol. Psychiatry.

[54] Ludwig-Körner C. (1999). Effects of Severely Disturbed Parents on Early Parent-Infant Interaction. Int. Forum Psychoanal..

[55] Opendak M., Robinson-Drummer P., Blomkvist A., Zanca R.M., Wood K., Jacobs L., Kirschner E. (2019). Neurobiology of maternal regulation of infant fear: The role of mesolimbic dopamine and its disruption by maltreatment. Neuropsychopharmacology.

[56] Uvnäs-Moberg K., Prime D.K. (2013). Oxytocin effects in mothers and infants during breastfeeding. Infant.

[57] Bowlby J. (1980). Loss: Sadness and Depression, Attachment and Loss.

[58] De Jaegher H., Di Paolo E.A., Morganti F., Carassa A., Riva G. (2008). Making sense in participation: An enactive approach to social cognition. Enacting Intersubjectivity: A Cognitive and Social Perspective to the Study of Interactions.

[59] Fuchs T., De Jaegher H. (2009). Enactive intersubjectivity: Participatory sense–making and mutual incorporation. Phenomenol. Cogn. Sci..

[60] Watkins J.G. (1954). Trance and transference. J. Clin. Exp. Hypn..

[61] Magai C., Cassidy J., Shaver P.R. (1999). Affect, Imagery and Attachment in Handbook of Attachment. Theory, Research and Clinical Applications.

[62] Scheff T.J. (1997). Emotions, the Social Bond and Human Reality.

[63] Baker E.L., Nash M.R., Nash M.R., Barnier A.J. (2008). Psychoanalytic approaches to clinical hypnosis. The Oxford Handbook of Hypnosis.

[64] Baker E.L. (2000). Reflections on the hypnotic relationship: Projective identification, containment, and attunement. Int. J. Clin. Exp. Hypn..

[65] Hari R., Kujala M.V. (2009). Brain Basis of Human Social Interaction: From Concepts to Brain Imaging. Physiol. Rev..

